# Nerve growth factor is primarily produced by GABAergic neurons of the adult rat cortex

**DOI:** 10.3389/fncel.2014.00220

**Published:** 2014-08-07

**Authors:** Jeremy Biane, James M. Conner, Mark H. Tuszynski

**Affiliations:** ^1^Department of Neurosciences, University of California at San DiegoLa Jolla, CA, USA; ^2^Veterans Affairs Medical CenterSan Diego, CA, USA

**Keywords:** nerve growth factor, basal forebrain, acetylcholine, plasticity, GABAergic, interneuron, protein expression and localization

## Abstract

Within the cortex, nerve growth factor (NGF) mediates the innervation of cholinergic neurons during development, maintains cholinergic corticopetal projections during adulthood and modulates cholinergic function through phenotypic control of the cholinergic gene locus. Recent studies suggest NGF may also play an important role in cortical plasticity in adulthood. Previously, NGF-producing cells have been shown to colocalize with GABAergic cell markers within the hippocampus, striatum, and basal forebrain. Classification of cells producing NGF in the cortex is lacking, however, and cholinergic corticopetal projections have been shown to innervate both pyramidal and GABAergic neurons in the cortex. In order to clarify potential trophic interactions between cortical neurons and cholinergic projections, we used double-fluorescent immunohistochemistry to classify NGF-expressing cells in several cortical regions, including the prefrontal cortex, primary motor cortex, parietal cortex and temporal cortex. Our results show that NGF colocalizes extensively with GABAergic cell markers in all cortical regions examined, with >91% of NGF-labeled cells coexpressing GAD65/67. Conversely, NGF-labeled cells exhibit very little co-localization with the excitatory cell marker CaMKIIα (<5% of cells expressing NGF). NGF expression was present in 56% of GAD-labeled cells, suggesting that production is confined to a specific subset of GABAergic neurons. These findings demonstrate that GABAergic cells are the primary source of NGF production in the cortex, and likely support the maintenance and function of basal forebrain cholinergic projections in adulthood.

## INTRODUCTION

The neurotrophin nerve growth factor (NGF) is involved in several critical processes in the developing and mature mammalian nervous system, including target innervation, cell differentiation and neuronal survival (Large et al., 1986; Li et al., 1995; Sofroniew et al., 2001; Lad et al., 2003). In the adult brain, NGF plays a key role in the maintenance and function of the basal forebrain cholinergic system (Lad et al., 2003). Levels of NGF are highest in areas receiving the greatest number of basal forebrain cholinergic projections, namely the hippocampus and cortex (Korsching et al., 1985; Shelton and Reichardt, 1986), and receptors for NGF in the cortex are largely confined to basal forebrain corticopetal projections (Kordower et al., 1988; Sofroniew et al., 2001; Rossi et al., 2002). Nerve growth factor signaling maintains cholinergic corticopetal projections during adulthood (Chen et al., 1997; Debeir et al., 1999) and can prevent degeneration of these neurons following transection of their axons (Hefti, 1986; Tuszynski et al., 1990; Kordower et al., 1994). Furthermore, NGF enhances release of acetylcholine from basal forebrain cultures (Auld et al., 2001) and can modulate cholinergic function through phenotypic control of the cholinergic gene locus (Rylett et al., 1993; Hu et al., 1997).

The basal forebrain cholinergic system influences learning and experience-dependent plasticity in the cortex (Bakin and Weinberger, 1996; Kilgard and Merzenich, 1998; Conner et al., 2003, 2005), and NGF signaling has been hypothesized to support, and possibly enhance, basal forebrain-mediated learning and plasticity. For example, blockade of NGF signaling via repeated injections of NGF antibodies in the insular cortex reduces local cholinergic innervation and disrupts acquisition of two processes that depend on the integrity of cholinergic inputs, conditioned taste aversion and inhibitory avoidance learning (Gutierrez et al., 1997). A recent study by Conner et al. (2009) demonstrated that both spatial learning and hippocampal long-term potentiation (LTP) are enhanced following infusions of NGF into the septal nucleus, the primary source of cholinergic innervation to the hippocampus.

Focal application of NGF can also induce rapid expansion of the local whisker representation in the barrel cortex of rats. This process is dependent on cholinergic projections from the basal forebrain (Prakash et al., 1996, 2004), suggesting that cortical NGF signaling can stimulate rapid cholinergic-dependent functional reorganization. Thus, in addition to its long-term trophic effects on basal forebrain cholinergic cells, NGF may also participate in short-term circuit remodeling. This “dual-action” hypothesis is supported by the existence of constitutive and activity-dependent release mechanisms of NGF (Blochl and Thoenen, 1995; Lessmann et al., 2003), potentially underlying the long-term trophic and short-term modulatory effects of NGF, respectively. Moreover, both retrograde (long-term) and local (short-term) signaling pathways have been observed for NGF (Huang and Reichardt, 2003).

Cells expressing NGF have been detected in the cortex, cerebellum, hippocampus, thalamus, striatum, basal forebrain and brainstem (Gall and Isackson, 1989; Maisonpierre et al., 1990; Isackson et al., 1991; Conner and Varon, 1992; Hayashi et al., 1993; Mufson et al., 1994; Zhang et al., 2007). Although NGF mRNA has been reportedly observed in astrocytes and other glia, the vast majority of cortical NGF is produced by neurons (Sofroniew et al., 2001). The specific types of neurons that produce NGF in the cortex are not known, however, and cholinergic corticopetal projections have been shown to innervate both excitatory and inhibitory neurons in the cortex (Zaborszky et al., 1999). In extracortical regions such as the hippocampus, striatum and basal forebrain, NGF production is predominantly localized to GABAergic inhibitory neurons (Lauterborn et al., 1993, 1995; Pascual et al., 1998; Bizon et al., 1999). Identification of NGF-producing cells in the cortex is important for identifying potential mechanisms underlying modulation of cholinergic cortical inputs, and mechanisms of cortical plasticity. In the current study, we identify a subset of cortical GABAergic cells as the primary source of NGF production in the adult cortex, with relatively rare production by glutamatergic neurons.

## MATERIALS AND METHODS

All subjects were treated in accordance with institutional guidelines for animal care. Due to low endogenous levels of cortical NGF expression that prohibited identification of specific cell–type sources of NGF in previous studies, we performed local colchicine infusions to inhibit microtubule polymerization, thereby blocking NGF transport away from the cell body and resulting in accumulation of detectable NGF antigen in the soma of producing cells (Schubert et al., 1972; Hokfelt et al., 1975; Hanson and Edström, 1978; Conner and Varon, 1992). Previous reports have shown that colchicine treatment increases detection of NGF using immunohistochemical techniques (Conner and Varon, 1992). Furthermore, colchicine levels similar to that used in the current study do not to induce gross abnormalities in neuronal distribution of the neurotrophin family member BDNF (Conner et al., 1997).

### TISSUE PREPARATION

Eight adult Fischer 344 rats (4 male, 4 female; Harlan Sprague Dawley) were anesthetized with 2 ml/kg of a 25 mg/ml ketamine, 1.3 mg/ml xylazine, and 0.25 mg/ml acepromazine cocktail, and injected with 5.0 μl colchicine (10 μg/μl in aCSF) at the cortical locations listed below at a rate of 0.5 μl/min using a 10 μl Hamilton syringe. Following infusion, the needle remained in place for four minutes to allow adequate diffusion to the surrounding tissue. Four animals received bilateral injections in the primary motor cortex (M1) at +1.2 mm anterior (A/P) and ±2.5 mm lateral (M/L) to bregma. Half the solution was injected at 1.6 mm below the surface of the brain (D/V), and the rest at 1.0 mm. The remaining four animals all received unilateral injections in the prefrontal cortex (A/P: +3.0, M/L: +0.5, D/V: -2.3 and -1.8), temporal cortex (A/P: -6.5, M/L: +5.0, D/V: -7.5 and -7.0), and parietal cortex (A/P: -3.0, M/L: +5.0, D/V: -1.8 and -1.3). After 48 h, animals were deeply anesthetized and transcardially perfused with 250 ml cold phosphate buffered saline (pH 7.4), followed by 250 ml of cold 2% paraformaldehyde + 0.2% parabenzoquinone in 0.1 M phosphate buffer. Brains were extracted, postfixed for 2 h in the same fixative, and cryoprotected in 0.1 M phosphate buffer containing 30% sucrose for at least 72 h at 4°C. Coronal sections (40 μm) were cut on a freezing sliding microtome and stored in cryoprotectant (TCS) at 4°C until further processed for immunohistochemistry.

### DOUBLE-LABEL FLUORESCENCE IMMUNOHISTOCHEMISTRY

Sequential double-label immunohistochemistry was used to visualize neurons expressing NGF and either GABAergic or glutamatergic cell markers. Free-floating sections were washed in Tris Buffered Saline (TBS), permeabilized with 0.25% Triton X-100, and nonspecific labeling was then blocked with 5% donkey serum. Sections were incubated for 72 h at 4°C in rabbit anti-NGF antibody (Conner and Varon, 1992) diluted 1:1000 in TBS, 0.25% Triton X-100, and 5% donkey serum. Following primary antibody incubation, sections were incubated in donkey anti-rabbit biotin-conjugated IgG (1:200; Vector Laboratories, Burlingame, CA, USA). Tyramide signal amplification (TSA; PerkinElmer, Waltham, MA, USA) was applied to amplify the NGF signal, after which sections were washed in TBS and incubated in Alexa Fluor 488 or 594-conjugated streptavidin (Invitrogen, Carlsbad, CA, USA) diluted 1:200 for 3 h at 4°C. After a brief wash, sections were incubated in both mouse anti-glutamate decarboxylase (GAD) 65 (GAD-6, AntibodyRegistry:AB_528264,1:2000; Developmental Studies Hybridoma Bank, Iowa City, IA, USA) and mouse anti-GAD67 (AnitbodyRegistry: AB_2278725, 1:1500; Millipore, Temecula, CA, USA), or in mouse anti-parvalbumin (AntibodyRegistry: AB_2174013, 1:30000; Millipore, Temecula, CA, USA), mouse anti-calbindin-D-28K (AntibodyRegistry: AB_476894, 1:1500; Sigma-Aldrich, St. Louis, MO, USA), or mouse anti-Ca^2+^/calmodulin-dependent protein kinase IIα (CaMKIIα, AntibodyRegistry: AB_2067919, 1:1500; Millipore) for 72 h at 4°C. Finally, sections were washed, incubated in Alexa Fluor 594 or 488-conjugated donkey anti-mouse (Invitrogen) for 3 h at room temperature, washed again, mounted on glass slides, and coverslipped in Fluoromount-G (Southern Biotech, Birminghan, AL, USA). In order to maximize identification of GABAergic cells, GAD65 and GAD67 antibodies were co-incubated. A subset of sections was coverslipped in ProLong Gold with DAPI (Invitrogen) for visualization of cell nuclei.

### ANTIBODY CHARACTERIZATION

The NGF antibody used in this study is an affinity-purified polyclonal raised in rabbit against purified mouse NGF (Conner and Varon, 1992). The antibody recognizes purified mouse and recombinant human NGF but does not cross react with recombinant BDNF or NT-3 (Conner and Varon, 1996). Furthermore, the immunoreactive pattern of NGF expression in the rat brain closely matches that obtained from in-situ analysis in the rat brain (Conner and Varon, 1997).

The monoclonal antibody GAD-65 (Developmental Studies Hybridoma Bank, Gad-6) was produced by immunizing mice with GAD protein immunoaffinity-purified from rat brain. Western blot analysis of rat brain homogenates revealed the antibody selectively recognizes GAD-65 but not GAD-67 (Chang and Gottlieb, 1988). Additional studies have shown that the GAD-6 antibody recognizes an epitope located between amino acids 475–571 of the C-terminus of GAD-65 (Butler et al., 1993).

The GAD-67 mouse monoclonal (Millipore, MAB 5406, lot: 25010139) was raised against amino acid residues 4-101 of human GAD-67, and recognizes a single 67-kDa band on Western blot analysis of rat brain (manufacturer’s technical information). Preincubation of the antibody with a GST-GAD-67 fusion protein resulted in no immunopositive signal in the brain (Ito et al., 2007).

The mouse monoclonal anti-CaMKIIα (Millipore, MAB 8699, lot: LV1366080) specifically recognizes the alpha subunit of calcium/calmodulin-dependent protein kinase II. Western blot analysis demonstrates that the antibody identifies a single band of 50 kDa and recognizes both phosphorylated and unphosphorylated forms (Erondu and Kennedy, 1985).

Monoclonal anti-calbindin-D-28k (Sigma–Aldrich, C9848, lot: 088k4799) is derived from BALB/c mice immunized with purified bovine kidney calbindin-D-28k. Immunoblotting showed the antibody recognizes a 28-kDa band, and does not to react with similar molecules, such as calbindin-D-9K, calretinin, myosin light chain, and parvalbumin (manufacturer’s technical information). Preabsorption with a calbindin-D-27 kDa protein purified from chick and rat brains was shown to eliminate calbindin immunostaining in the brain (Pasteels et al., 1987).

Anti-parvalbumin (Millipore, MAB 1572, lot: LV1378387) was collected from mice immunized against parvalbumin purified from frog muscle. The monoclonal antibody is directed against an epitope at the first Ca^2+^-binding site and immunoblot analysis demonstrates it recognizes a brain protein of 12 kDa (manufacturer’s technical information).

### CONFOCAL ANALYSIS

Images were captured using an Olympus AX70 with Magnafire software (version 2.0; Karl Storz Imaging, Goleta, CA, USA). Because NGF labeling was reduced substantially approximately 2 mm anterior/posterior to colchicine injection sites, presumably due to a lack of colchicine diffusion and the resulting absence of somatic NGF accumulation, only sections within 1.5 mm of each colchicine injection site were analyzed. Every 10^th^ section (400 μm) was examined within a cortical region. Cells were manually counted, and at least three sections were analyzed in each cortical region and antibody combination per subject. Only three animals were evaluated in the temporal region due to undetectable NGF labeling in the fourth. Single and double-labeled cells were quantified using both single and double-channel images. Several criteria were used to identify labeled cells, including size, morphology, signal vs. background, and coincident DAPI labeling when assessing DAPI-stained tissue. In a subset of sections, 5 μm z-stacks were collected using an Olympus Fluoview FV1000 to ensure neuronal localization of the labeled object. Due to our interest in the proportion, and not the absolute number, of double-labeled cells, stereological methods were not used.

The percentage of double-labeled cells per immunoreactive (IR) cell group was determined for each image field. Mean ± standard error was calculated for each cortical region examined. One-way analysis of variance (ANOVA) was used to evaluate differences among cortical regions. Fisher’s HSD was used for post-hoc analysis. All statistical analyses were carried out with SPSS 15.0 for Windows.

### ANTIBODY CONTROLS

Controls included omission of primary antibodies, omission of secondary antibodies, and replacement of primary antibody with nonspecific antibody (rabbit IgG). All manipulations had the expected effects and supported the assertion that labeled cells represent true antigen labeling by their corresponding antibodies.

## RESULTS

### NGF IMMUNOLABELING

**FIGURE 1:**
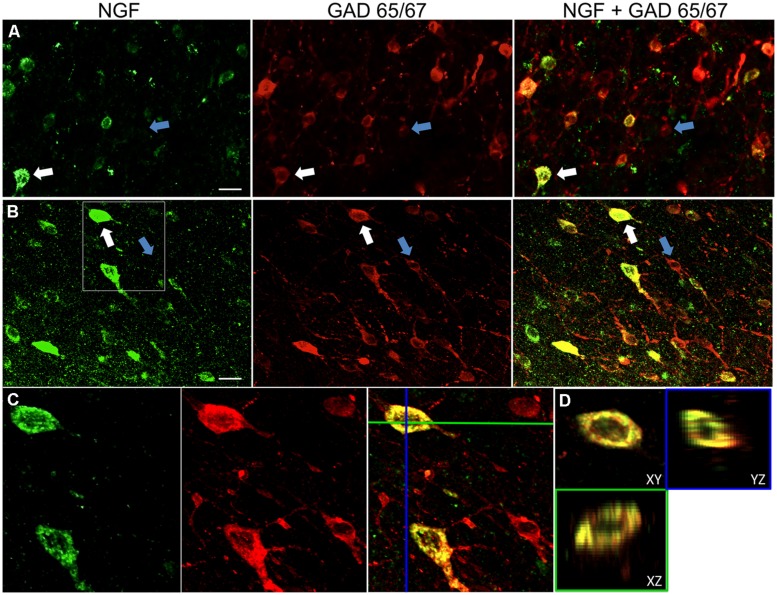
**Nerve growth factor colocalizes extensively with inhibitory cell markers.** Immunoreactive cells in the **(A)** primary motor cortex and **(B)** prefrontal cortex. Images show that NGF-labeled cells (green) colocalize extensively with GAD 65/67-labeled cells (red). White arrows show an example cell immunoreactive for both NGF and GAD 65/67 in each cortical region. Blue arrows show cells immunoreactive for GAD 65/67 only. **(C)** Magnified image of area inside white box in **(B)**. **(D)** *X*–*Y*–*Z* reconstruction of the double-labeled cell indicated in **(C)**. Green and Blue lines indicate dissection levels in *XZ* and *YZ* planes, respectively. Scale bars = 25 μm.

Distinct NGF labeling was visible within a radius of 1.5 mm from colchicine cortical injection sites. Within these areas, NGF labeling was predominantly confined to cell somata (**Figure 1**). Rarely, one or more cellular processes could also be distinguished. Outside of this 1.5 mm radius, NGF labeling was virtually undetectable in the cortex. As cortical expression of the NGF receptors TrkA and p75 are confined to cholinergic corticopetal fibers, the observed labeling of NGF is unlikely to reflect endocytosed NGF, but instead is indicative of NGF-producing cells (Holtzman et al., 1995; Rossi et al., 2002; Stephens et al., 2005). As reported previously (Ribak et al., 1978), colchicine treatment intensified GAD labeling in cell bodies as well as neuronal processes. Colchicine had no detectable effect on labeling for parvalbumin, calbindin, or CaMKIIα.

### NGF AND GABAergic CO-LOCALIZATION

**FIGURE 2:**
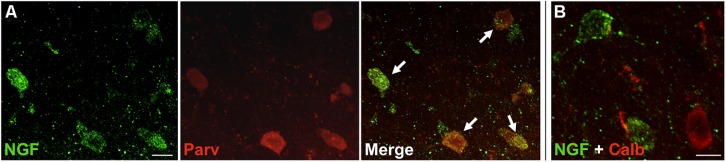
**NGF colabeled with inhibitory neuron subclass markers.** Slices of the motor cortex were labeled for NGF and either parvalbumin or calbindin. **(A)** Cells showed extensive overlap of NGF and parvalbumin labeling (white arrows). **(B)** Conversely, colabeling of NGF and calbindin was less common. Scale Bars = 25 μm.

Nerve growth factor co-localized extensively with the GABAergic cell markers GAD65 and GAD67, regardless of the cortical area examined (**Figure 1**; **Table 1**). Overall, 91 ± 0.9% of NGF-labeled cortical cells also labeled for GAD65/67. The percentage of NGF-labeled cells co-expressing GAD65/67 showed little difference among the prefrontal (90.0 ± 1.5%), motor (91.7 ± 1.5%), parietal (89.6 ± 3.5%) and temporal (93.4 ± 8.1%) cortices (one-way ANOVA; *p* = 0.78). Conversely, NGF co-localized with only 55 ± 2.3% of all GAD65/67-labeled cells. To determine if NGF production was restricted to a specific subtype of GABAergic neuron, we co-labeled tissue for NGF and either parvalbumin or calbindin (**Figure 2**). NGF-labeled cells were observed to colocalize with both markers. However, NGF colocalization with parvalbumin (67.8 ± 3.6%) was over 2× greater than with calbindin (29.1 ± 3.9%). Additionally, NGF-IR cells constituted less than half of all parvalbumin (47.7 ± 4.6%) and calbindin (25.7 ± 4.9%) immunoreactive cells.

**Table 1:** NGF- and GAD65/67-immunoreactive cells by cortical region.

Cortical region	NGF I.R. cells	GAD65/67 I.R. cells	Double I.R. cells	% NGF double I.R.	% GAD65/67 double I.R.
Prefrontal	180	312	163	90.0 ± 1.5	52.2 ± 1.6
Motor	530	830	486	91.7 ± 1.5	58.5 ± 2.2
Parietal	134	247	120	89.6 ± 3.5	48.6 ± 5.1
Temporal	61	100	57	93.4 ± 8.1	57.0 ± 7.3

Total	905	1489	826	**91.3**	55.5

Nerve growth factor-expressing neurons were observed throughout all cortical layers. Previous studies have reported uneven distribution of NGF-labeled neurons in the cortical laminae (Pitts and Miller, 2000; Patz and Wahle, 2006). Quantitative analysis by layer was not performed in the current study, however, as NGF labeling intensity diminished with increasing distance from the colchicine injection site.

### NGF AND GLUTAMATERGIC CO-LOCALIZATION

**FIGURE 3:**
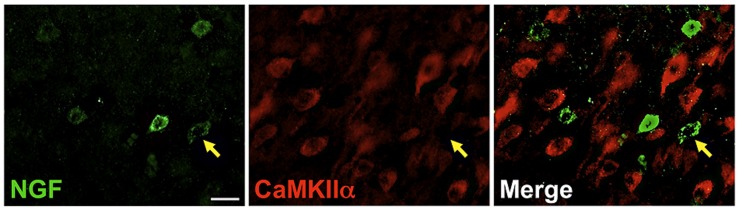
**Nerve growth factor colocalizes minimally with the excitatory cell marker CaMKIIα.** Immunoreactive cells in the prefrontal cortex. Cells were rarely colabeled for NGF (green) and CaMKIIα (red), regardless of cortical region examined. Gold arrows show an example of a cell immunoreactive for NGF only. Scale bars = 25 μm.

Labeling for CaMKIIα was primarily observed within cell somata and proximal processes (**Figure 3**). Unlike the extensive co-localization seen with NGF and GABAergic markers, NGF-labeled cells rarely co-localized with CaMKIIα-labeled cells (**Figure 3**; **Table 2**). In total, 4.9 ± 1.1% of NGF-immunoreactive cells were co-labeled with CaMKIIα antibodies. Co-localization differed significantly by cortical region (one-way ANOVA; *p* = 0.03); Fisher’s *post hoc* revealed that the prefrontal cortex had a greater proportion of double-labeled NGF cells (7.6 ± 2.1%) compared to the primary motor cortex (2.4 ± 1.0%; *p* = 0.02) and parietal cortex (2.9% ± 1.5; *p* = 0.01).

**Table 2:** NGF- and CaMKIIα-immunoreactive cells by cortical region.

Cortical region	NGF I.R. cells	CaMKIIa I.R. cells	Double I.R. cells	% NGF double I.R.	% CaMKIIa double I.R.
Prefrontal	317	649	24	7.6 ± 2.1	3.7 ± 1.1
Motor	125	380	3	2.4 ± 1.0	0.8 ± 0.3
Parietal	240	508	7	2.9 ± 1.5	1.4 ± 0.6
Temporal	182	519	8	4.4 ± 1.6	1.5 ± 0.6

Total	864	2056	42	4.9	2.0

Cells immunoreactive for CaMKIIα greatly outnumbered those labeled by NGF antibodies. The overall proportion of CaMKIIα-labeled cells simultaneously expressing NGF signal was 2 ± 0.6%. This percentage differed significantly by region (one-way ANOVA *p* = 0.003), with the prefrontal cortex exhibiting a greater proportion of double-labeled NGF/CaMKIIα cells (3.7 ± 1.1%) than the primary motor cortex (0.8% ± 0.3; *p* = 0.001), the parietal cortex (1.4% ± 0.6; *p* = 0.01), and the temporal cortex (1.5 ± 0.6%; *p* = 0.01).

## DISCUSSION

The current study demonstrates that the vast majority (>90%) of NGF-producing neurons of the cortex are GABAergic, while half of all GABAergic neurons colocalize with NGF. In contrast, markers of excitatory neurons exhibit only rare co-localization with NGF. These results were consistent throughout multiple cortical regions analyzed in this study, indicating that NGF is primarily produced by inhibitory interneurons in the rat neocortex.

Although NGF immunoreactivity rarely coincided with excitatory cell markers (CAMKIIα), a small percentage (~5%) were positive for CaMKIIα throughout all examined cortical regions. Confocal analysis confirmed that this double labeling originated from the same focal plane, and was not due to discrete signal arising from overlapping cells. The functional significance of NGF expression in such a small fraction of excitatory cells in not known but it is possible that these NGF-producing neurons represent a previously unidentified subclass of excitatory neurons in the neocortex.

Our results are consistent with findings of earlier studies in other brain regions demonstrating that NGF co-localizes almost exclusively with GABAergic cells in the striatum, basal forebrain, and hippocampus (Lauterborn et al., 1993, 1995; Pascual et al., 1998; Bizon et al., 1999). Thus, NGF production by GABAergic cells may be a general property of all targets receiving basal forebrain cholinergic innervation (including the basal forebrain itself). These cholinergic neuronal populations require NGF for maintenance of their phenotype and projections (Rylett et al., 1993; Chen et al., 1997; Hu et al., 1997; Debeir et al., 1999). Across studies, inhibitory interneurons now emerge as the primary source of NGF trophism for basal forebrain cholinergic neurons.

Although basal forebrain cholinergic neurons innervate both excitatory and inhibitory cortical networks (Zaborszky et al., 1999), the source of trophic support is overwhelmingly from the inhibitory population, which is known to play a critical role in mediating plasticity in cortical circuits (Hensch and Stryker, 2004; Yazaki-Sugiyama et al., 2009; Donato et al., 2013). Cholinergic signaling appears to contribute to the differential activation of various inhibitory subpopulations, thereby modulating excitatory-inhibitory balance (Xiang et al., 1998; Froemke et al., 2007). NGF may thereby serve as a feedback signal between highly plastic inhibitory networks and the cholinergic inputs that activate them. Indeed, NGF augments plasticity and behavioral learning through cholinergic-dependent mechanisms (Prakash et al., 1996, 2004; Gutierrez et al., 1997; Conner et al., 2009). Activity-dependent release of NGF (Blochl and Thoenen, 1995; Lessmann et al., 2003), paired with the ability of NGF to increase cholinergic activity (Rylett et al., 1993; Hu et al., 1997; Auld et al., 2001), suggests that NGF may promote reorganization of active circuits via enhanced cholinergic function.

Future studies will attempt to identify which subclasses of inhibitory neurons express NGF and how this expression precisely influences cortical circuitry. To this end, we took preliminary steps to determine whether NGF co-localized with two common markers of inhibitory neurons, parvalbumin (primarily associated with basket and chandelier cells), and calbindin (associated with numerous inhibitory cell types; McBain and Fisahn, 2001; Markram et al., 2004). Our results demonstrate that NGF was primarily detected in parvalbumin-immunoreactive cells, although many cells labeled by parvalbumin did not co-express NGF. Importantly, NGF-IR cells also colocalized with calbindin-labeled cells, suggesting that more than one subclass of inhibitory neuron produces NGF.

In summary, within the rat neocortex NGF is primarily expressed by inhibitory neurons, a property that appears to be conserved from phylogenetically older brain areas and which may generalize to the brain as a whole. Our results suggest cortical inhibitory interneurons play a vital role in the maintenance of cholinergic projection neurons of the basal forebrain. GABAergic interneurons thus may promote cortical reorganization via regulated NGF signaling.

## Conflict of Interest Statement

The authors declare that the research was conducted in the absence of any commercial or financial relationships that could be construed as a potential conflict of interest.

## References

[B1] Auld D. S. Mennicken F. Quirion R. (2001). Nerve growth factor rapidly induces prolonged acetylcholine release from cultured basal forebrain neurons: differentiation between neuromodulatory and neurotrophic influences. *J. Neurosci.* 21 3375–3382 11331367 10.1523/JNEUROSCI.21-10-03375.2001 PMC6762468

[B2] Bakin J. S. Weinberger N. M. (1996). Induction of a physiological memory in the cerebral cortex by stimulation of the nucleus basalis. *Proc. Natl. Acad. Sci. U.S.A.* 93 11219–11224 10.1073/pnas.93.20.11219 8855336 PMC38311

[B3] Bizon J. L. Lauterborn J. C. Gall C. M. (1999). Subpopulations of striatal interneurons can be distinguished on the basis of neurotrophic factor expression. *J. Comp. Neurol.* 408 283–298 10.1002/(SICI)1096-9861(19990531)408:2<283::AID-CNE9>3.0.CO;2-2 10333275

[B4] Blochl A. Thoenen H. (1995). Characterization of nerve growth factor (NGF) release from hippocampal neurons: evidence for a constitutive and an unconventional sodium-dependent regulated pathway. *Eur. J. Neurosci.* 7 1220–1228 10.1111/j.1460-9568.1995.tb01112.x 7582095

[B5] Butler M. H. Solimena M. Dirkx R. Jr. Hayday A. De Camilli P. (1993). Identification of a dominant epitope of glutamic acid decarboxylase (GAD-65) recognized by autoantibodies in stiff-man syndrome. *J. Exp. Med.* 178 2097–2106 10.1084/jem.178.6.2097 8245784 PMC2191306

[B6] Chang Y. C. Gottlieb D. I. (1988). Characterization of the proteins purified with monoclonal antibodies to glutamic acid decarboxylase. *J. Neurosci.* 8 2123–2130 3385490 10.1523/JNEUROSCI.08-06-02123.1988 PMC6569335

[B7] Chen K. S. Nishimura M. C. Armanini M. P. Crowley C. Spencer S. D. Phillips H. S. (1997). Disruption of a single allele of the nerve growth factor gene results in atrophy of basal forebrain cholinergic neurons and memory deficits. *J. Neurosci.* 17 7288–7296 9295375 10.1523/JNEUROSCI.17-19-07288.1997 PMC6573440

[B8] Conner J. M. Chiba A. A. Tuszynski M. H. (2005). The basal forebrain cholinergic system is essential for cortical plasticity and functional recovery following brain injury. *Neuron* 46 173–179 10.1016/j.neuron.2005.03.003 15848797

[B9] Conner J. M. Culberson A. Packowski C. Chiba A. A. Tuszynski M. H. (2003). Lesions of the Basal forebrain cholinergic system impair task acquisition and abolish cortical plasticity associated with motor skill learning. *Neuron* 38 819–829 10.1016/S0896-6273(03)00288-5 12797965

[B10] Conner J. M. Franks K. M. Titterness A. K. Russell K. Merrill D. A. Christie B. R. (2009). NGF is essential for hippocampal plasticity and learning. *J. Neurosci.* 29 10883–10889 10.1523/JNEUROSCI.2594-09.2009 19726646 PMC2765804

[B11] Conner J. M. Lauterborn J. C. Yan Q. Gall C. M. Varon S. (1997). Distribution of brain-derived neurotrophic factor (BDNF) protein and mRNA in the normal adult rat CNS: evidence for anterograde axonal transport. *J. Neurosci.* 17 2295–2313 9065491 10.1523/JNEUROSCI.17-07-02295.1997 PMC6573520

[B12] Conner J. M. Varon S. (1992). Distribution of nerve growth factor-like immunoreactive neurons in the adult rat brain following colchicine treatment. *J. Comp. Neurol.* 326 347–362 10.1002/cne.903260304 1469118

[B13] Conner J. M. Varon S. (1996). Characterization of antibodies to nerve growth factor: assay-dependent variability in the cross-reactivity with other neurotrophins. *J. Neurosci. Methods* 65 93–99 10.1016/0165-0270(95)00151-4 8815313

[B14] Conner J. M. Varon S. (1997). Developmental profile of NGF immunoreactivity in the rat brain: a possible role of NGF in the establishment of cholinergic terminal fields in the hippocampus and cortex. *Brain Res. Dev. Brain Res.* 101 67–79 10.1016/S0165-3806(97)00051-5 9263581

[B15] Debeir T. Saragovi H. U. Cuello A. C. (1999). A nerve growth factor mimetic TrkA antagonist causes withdrawal of cortical cholinergic boutons in the adult rat. *Proc. Natl. Acad. Sci. U.S.A.* 96 4067–4072 10.1073/pnas.96.7.4067 10097164 PMC22421

[B16] Donato F. Rompani S. B. Caroni P. (2013). Parvalbumin-expressing basket-cell network plasticity induced by experience regulates adult learning. *Nature* 504 272–276 10.1038/nature12866 24336286

[B17] Erondu N. E. Kennedy M. B. (1985). Regional distribution of type II Ca2+/calmodulin-dependent protein kinase in rat brain. *J. Neurosci.* 5 3270–3277 4078628 10.1523/JNEUROSCI.05-12-03270.1985 PMC6565219

[B18] Froemke R. C. Merzenich M. M. Schreiner C. E. (2007). A synaptic memory trace for cortical receptive field plasticity. *Nature* 450 425–429 10.1038/nature06289 18004384

[B19] Gall C. M. Isackson P. J. (1989). Limbic seizures increase neuronal production of messenger RNA for nerve growth factor. *Science* 245 758–761 10.1126/science.2549634 2549634

[B20] Gutierrez H. Miranda M. I. Bermudez-Rattoni F. (1997). Learning impairment and cholinergic deafferentation after cortical nerve growth factor deprivation. *J. Neurosci.* 17 3796–3803 9133398 10.1523/JNEUROSCI.17-10-03796.1997 PMC6573716

[B21] Hanson M. Edström A. (1978). Mitosis inhibitors and axonal transport. *Int. Rev. Cytol. Suppl.* 373–402 79566

[B22] Hayashi M. Yamashita A. Shimizu K. Sogawa K. Fujii Y. (1993). Expression of the gene for nerve growth factor (NGF) in the monkey central nervous system. *Brain Res.* 618 142–148 10.1016/0006-8993(93)90437-R 8402167

[B23] Hefti F. (1986). Nerve growth factor promotes survival of septal cholinergic neurons after fimbrial transections. *J. Neurosci.* 6 2155–2162 3746405 10.1523/JNEUROSCI.06-08-02155.1986 PMC6568758

[B24] Hensch T. K. Stryker M. P. (2004). Columnar architecture sculpted by GABA circuits in developing cat visual cortex. *Science* 303 1678–1681 10.1126/science.1091031 15017001 PMC2562723

[B25] Hokfelt T. Kellerth J. O. Nilsson G. Pernow B. (1975). Experimental immunohistochemical studies on the localization and distribution of substance P in cat primary sensory neurons. *Brain Res.* 100 235–252 10.1016/0006-8993(75)90481-3 1104079

[B26] Holtzman D. M. Kilbridge J. Li Y. Cunningham E. T. Jr. Lenn N. J. Clary D. O. (1995). TrkA expression in the CNS: evidence for the existence of several novel NGF-responsive CNS neurons. *J. Neurosci.* 15 1567–1576 7869118 10.1523/JNEUROSCI.15-02-01567.1995 PMC2710116

[B27] Hu L. Cote S. L. Cuello A. C. (1997). Differential modulation of the cholinergic phenotype of the nucleus basalis magnocellularis neurons by applying NGF at the cell body or cortical terminal fields. *Exp. Neurol.* 143 162–171 10.1006/exnr.1996.6357 9000455

[B28] Huang E. J. Reichardt L. F. (2003). Trk receptors: roles in neuronal signal transduction. *Annu. Rev. Biochem.* 72 609–642 10.1146/annurev.biochem.72.121801.161629 12676795

[B29] Isackson P. J. Huntsman M. M. Murray K. D. Gall C. M. (1991). BDNF mRNA expression is increased in adult rat forebrain after limbic seizures: temporal patterns of induction distinct from NGF. *Neuron* 6 937–948 10.1016/0896-6273(91)90234-Q 2054188

[B30] Ito T. Hioki H. Nakamura K. Tanaka Y. Nakade H. Kaneko T. (2007). Gamma-aminobutyric acid-containing sympathetic preganglionic neurons in rat thoracic spinal cord send their axons to the superior cervical ganglion. *J. Comp. Neurol.* 502 113–125 10.1002/cne.21309 17335042

[B31] Kilgard M. P. Merzenich M. M. (1998). Cortical map reorganization enabled by nucleus basalis activity. *Science* 279 1714–1718 10.1126/science.279.5357.1714 9497289

[B32] Kordower J. H. Bartus R. T. Bothwell M. Schatteman G. Gash D. M. (1988). Nerve growth factor receptor immunore activity in the nonhuman primate (Cebus apella): distribution, morphology, and colocalization with cholinergic enzymes. *J. Comp. Neurol.* 277 465–486 10.1002/cne.902770402 2850304

[B33] Kordower J. H. Winn S. R. Liu Y. T. Mufson E. J. Sladek J. R. Jr. Hammang J. P. (1994). The aged monkey basal forebrain: rescue and sprouting of axotomized basal forebrain neurons after grafts of encapsulated cells secreting human nerve growth factor. *Proc. Natl. Acad. Sci. U.S.A.* 91 10898–10902 10.1073/pnas.91.23.10898 7971980 PMC45133

[B34] Korsching S. Auburger G. Heumann R. Scott J. Thoenen H. (1985). Levels of nerve growth factor and its mRNA in the central nervous system of the rat correlate with cholinergic innervation. *EMBO J.* 4 1389–1393 2411537 10.1002/j.1460-2075.1985.tb03791.x PMC554356

[B35] Lad S. P. Neet K. E. Mufson E. J. (2003). Nerve growth factor: structure, function and therapeutic implications for Alzheimer’s disease. *Curr. Drug Targets CNS Neurol. Disord.* 2 315–334 10.2174/1568007033482724 14529363

[B36] Large T. H. Bodary S. C. Clegg D. O. Weskamp G. Otten U. Reichardt L. F. (1986). Nerve growth factor gene expression in the developing rat brain. *Science* 234 352–355 10.1126/science.3764415 3764415

[B37] Lauterborn J. C. Bizon J. L. Tran T. M. Gall C. M. (1995). NGF mRNA is expressed by GABAergic but not cholinergic neurons in rat basal forebrain. *J. Comp. Neurol.* 360 454–462 10.1002/cne.903600307 8543651

[B38] Lauterborn J. C. Tran T. M. Isackson P. J. Gall C. M. (1993). Nerve growth factor mRNA is expressed by GABAergic neurons in rat hippocampus. *Neuroreport* 5 273–276 10.1097/00001756-199312000-00023 8298089

[B39] Lessmann V. Gottmann K. Malcangio M. (2003). Neurotrophin secretion: current facts and future prospects. *Prog. Neurobiol.* 69 341–374 10.1016/S0301-0082(03)00019-4 12787574

[B40] Li Y. Holtzman D. M. Kromer L. F. Kaplan D. R. Chua-Couzens J. Clary D. O. (1995). Regulation of TrkA and ChAT expression in developing rat basal forebrain: evidence that both exogenous and endogenous NGF regulate differentiation of cholinergic neurons. *J. Neurosci.* 15 2888–2905 7536822 10.1523/JNEUROSCI.15-04-02888.1995 PMC6577746

[B41] Maisonpierre P. C. Belluscio L. Friedman B. Alderson R. F. Wiegand S. J. Furth M. E. (1990). NT-3, BDNF, and NGF in the developing rat nervous system: parallel as well as reciprocal patterns of expression. *Neuron* 5 501–509 10.1016/0896-6273(90)90089-X 1688327

[B42] Markram H. Toledo-Rodriguez M. Wang Y. Gupta A. Silberberg G. Wu C. (2004). Interneurons of the neocortical inhibitory system. *Nat. Rev. Neurosci.* 5 793–807 10.1038/nrn1519 15378039

[B43] McBain C. J. Fisahn A. (2001). Interneurons unbound. *Nat. Rev. Neurosci.* 2 11–23 10.1038/35049047 11253355

[B44] Mufson E. J. Conner J. M. Varon S. Kordower J. H. (1994). Nerve growth factor-like immunoreactive profiles in the primate basal forebrain and hippocampal formation. *J. Comp. Neurol.* 341 507–519 10.1002/cne.903410407 8201026

[B45] Pascual M. Rocamora N. Acsady L. Freund T. F. Soriano E. (1998). Expression of nerve growth factor and neurotrophin-3 mRNAs in hippocampal interneurons: morphological characterization, levels of expression, and colocalization of nerve growth factor and neurotrophin-3. *J. Comp. Neurol.* 395 73–90 10.1002/(SICI)1096-9861(19980525)395:1<73::AID-CNE6>3.0.CO;2-E 9590547

[B46] Pasteels B. Miki N. Hatakenaka S. Pochet R. (1987). Immunohistochemical cross-reactivity and electrophoretic comigration between calbindin D-27 kDa and visinin. *Brain Res.* 412 107–113 10.1016/0006-8993(87)91444-2 3607443

[B47] Patz S. Wahle P. (2006). Developmental changes of neurotrophin mRNA expression in the layers of rat visual cortex. *Eur. J. Neurosci.* 24 2453–2460 10.1111/j.1460-9568.2006.05126.x 17100834

[B48] Pitts A. F. Miller M. W. (2000). Expression of nerve growth factor, brain-derived neurotrophic factor, and neurotrophin-3 in the somatosensory cortex of the mature rat: coexpression with high-affinity neurotrophin receptors. *J. Comp. Neurol.* 418 241–254 10.1002/(SICI)1096-9861(20000313)418:3<241::AID-CNE1>3.0.CO;2-M 10701824

[B49] Prakash N. Cohen-Cory S. Frostig R. D. (1996). RAPID and opposite effects of BDNF and NGF on the functional organization of the adult cortex in vivo. *Nature* 381 702–706 10.1038/381702a0 8649516

[B50] Prakash N. Cohen-Cory S. Penschuck S. Frostig R. D. (2004). Basal forebrain cholinergic system is involved in rapid nerve growth factor (NGF)-induced plasticity in the barrel cortex of adult rats. *J. Neurophysiol.* 91 424–437 10.1152/jn.00489.2003 14507983

[B51] Ribak C. E. Vaughn J. E. Saito K. (1978). Immunocytochemical localization of glutamic acid decarboxylase in neuronal somata following colchicine inhibition of axonal transport. *Brain Res.* 140 315–332 10.1016/0006-8993(78)90463-8 75042

[B52] Rossi F. M. Sala R. Maffei L. (2002). Expression of the nerve growth factor receptors TrkA and p75NTR in the visual cortex of the rat: development and regulation by the cholinergic input. *J. Neurosci.* 22 912–919 11826120 10.1523/JNEUROSCI.22-03-00912.2002 PMC6758534

[B53] Rylett R. J. Goddard S. Schmidt B. M. Williams L. R. (1993). Acetylcholine synthesis and release following continuous intracerebral administration of NGF in adult and aged Fischer-344 rats. *J. Neurosci.* 13 3956–3963 8366354 10.1523/JNEUROSCI.13-09-03956.1993 PMC6576469

[B54] Schubert P. Kreutzberg G. W. Lux H. D. (1972). Neuroplasmic transport in dendrites: effect of colchicine on morphology and physiology of motoneurones in the cat. *Brain Res.* 47 331–343 10.1016/0006-8993(72)90643-9 4118532

[B55] Shelton D. L. Reichardt L. F. (1986). Studies on the expression of the beta nerve growth factor (NGF) gene in the central nervous system: level and regional distribution of NGF mRNA suggest that NGF functions as a trophic factor for several distinct populations of neurons. *Proc. Natl. Acad. Sci. U.S.A.* 83 2714–2718 10.1073/pnas.83.8.2714 3458230 PMC323370

[B56] Sofroniew M. V. Howe C. L. Mobley W. C. (2001). Nerve growth factor signaling, neuroprotection, and neural repair. *Annu. Rev. Neurosci.* 24 1217–1281 10.1146/annurev.neuro.24.1.1217 11520933

[B57] Stephens H. E. Belliveau A. C. Gupta J. S. Mirkovic S. Kablar B. (2005). The role of neurotrophins in the maintenance of the spinal cord motor neurons and the dorsal root ganglia proprioceptive sensory neurons. *Int. J. Dev. Neurosci.* 23 613–620 10.1016/j.ijdevneu.2005.07.002 16183241

[B58] Tuszynski M. H. Armstrong D. M. Gage F. H. (1990). Basal forebrain cell loss following fimbria/fornix transection. *Brain Res.* 508 241–248 10.1016/0006-8993(90)90402-W 2306615

[B59] Xiang Z. Huguenard J. R. Prince D. A. (1998). Cholinergic switching within neocortical inhibitory networks. *Science* 281 985–988 10.1126/science.281.5379.985 9703513

[B60] Yazaki-Sugiyama Y. Kang S. Cateau H. Fukai T. Hensch T. K. (2009). Bidirectional plasticity in fast-spiking GABA circuits by visual experience. *Nature* 462 218–221 10.1038/nature08485 19907494

[B61] Zaborszky L. Pang K. Somogyi J. Nadasdy Z. Kallo I. (1999). The basal forebrain corticopetal system revisited. *Ann. N. Y. Acad. Sci.* 877 339–367 10.1111/j.1749-6632.1999.tb09276.x 10415658

[B62] Zhang H. T. Li L. Y. Zou X. L. Song X. B. Hu Y. L. Feng Z. T. (2007). Immunohistochemical distribution of NGF, BDNF, NT-3, and NT-4 in adult rhesus monkey brains. *J. Histochem. Cytochem.* 55 1–19 10.1369/jhc.6A6952.2006 16899765

