# Supplementary figures and images for: Nerve growth factor is primarily produced by GABAergic neurons of the adult rat cortex
Source: Front Cell Neurosci. 2014 Aug 7;8:220. doi: 10.3389/fncel.2014.00220 (PMC4124705; doi:10.3389/fncel.2014.00220)

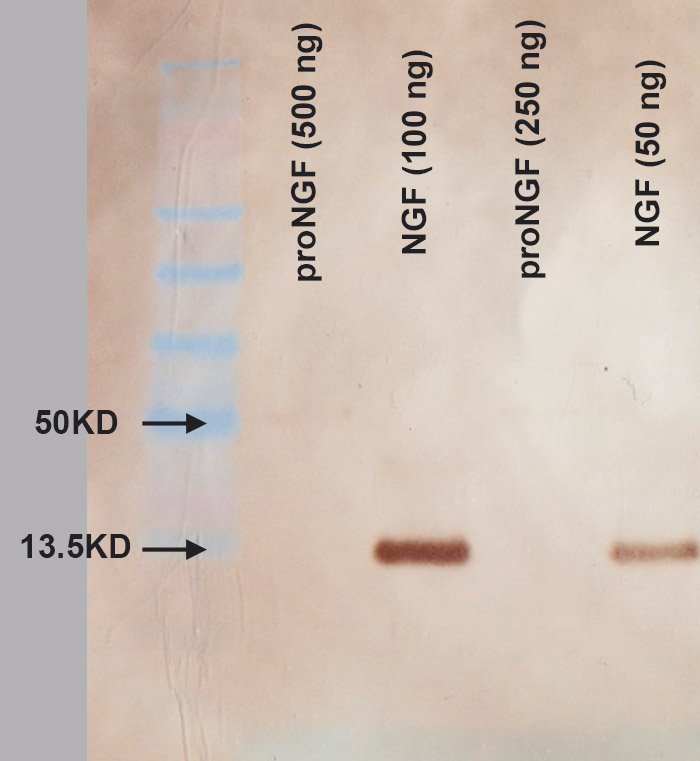

Supplement: Supplementary file 1 [file Image_1.JPEG]
